# Age-stratified trajectories of patient-reported outcomes and perioperative safety after robot-assisted radical prostatectomy: a prospective multicenter cohort study

**DOI:** 10.1038/s41598-026-46171-z

**Published:** 2026-03-31

**Authors:** Norihiko Kawamura, Masashi Nakayama, Yusuke Inagaki, Koichi Tsutahara, Koji Hatano, Jiro Nakayama, Yohei Okuda, Yosuke Sekii, Koji Yazawa, Tetsuya Takao, Norio Nonomura, Masao Tsujihata, Yasushi Miyagawa, Hitoshi Takayama, Akira Nagahara, Kazuo Nishimura

**Affiliations:** 1 Department of Urology, Osaka International Cancer Institute, 3-1-69 Otemae, Chuo-ku, Osaka, 541-8567 Japan; 2 Department of Urology, Osaka Keisatsu Hospital, Osaka, Japan; 3 Department of Urology, Osaka General Medical Center, Osaka, Japan; 4 Department of Urology, The University of Osaka Graduate School of Medicine, Suita, Japan; 5 Department of Urology, Osaka Rosai Hospital, Sakai, Japan; 6 Department of Urology, Sumitomo Hospital, Osaka, Japan; 7 Department of Urology, Sakai City Medical Center, Sakai, Japan

**Keywords:** Prostate cancer, Robot-assisted radical prostatectomy, Elderly patients, Quality of life, Patient-reported outcomes, Prospective cohort study, Cancer, Diseases, Medical research, Oncology, Urology

## Abstract

As life expectancy increases, a growing number of older adults undergo radical prostatectomy for localized prostate cancer; however, longitudinal age-specific functional outcomes remain insufficiently characterized. We conducted a prospective multicenter cohort study evaluating age-stratified trajectories of patient-reported outcomes (PROs) assessed using the Expanded Prostate Cancer Index Composite (EPIC) and perioperative complications following robot-assisted radical prostatectomy (RARP). A total of 604 patients were categorized into three age groups (< 65, 65–74, and ≥ 75 years). PROs were assessed using the EPIC at baseline and at 1, 3, 6, and 12 months postoperatively, and longitudinal changes were analyzed using linear mixed-effects models. Urinary function declined transiently after surgery in all age groups and substantially recovered by 12 months, with no significant differences between men aged ≥ 75 years and younger patients. Sexual function decreased irrespective of age, although younger patients maintained higher absolute scores after nerve-sparing procedures. In contrast, sexual bother demonstrated an age-dependent pattern, with minimal change observed in men aged ≥ 75 years. Perioperative complication rates did not differ significantly across age groups. These findings suggest that chronological age alone should not preclude consideration of RARP in appropriately selected elderly patients and provide evidence supporting individualized surgical decision-making in an aging population.

**Supplementary Information:**

The online version contains supplementary material available at 10.1038/s41598-026-46171-z.

## Introduction

Prostate cancer (PCa) is the second most commonly diagnosed cancer among men worldwide, with the highest incidence reported in North and South America, Europe, and Australia/New Zealand^1^. With the global increase in life expectancy, the proportion of older adults diagnosed with PCa has been increasing^2–4^. For example, life expectancy at birth of men in the United States increased from 74.2 years in 2000 to 76.5 years in 2019^5^. Similar trends are observed in European and East Asian countries, where life expectancy has extended even further in some cases. This demographic shift highlights the importance of effectively managing PCa in older men, a population characterized by a higher prevalence of comorbidities and frailty, which complicates treatment decision-making.

Treatment options for older men with localized PCa include active surveillance, radiation therapy, and radical prostatectomy. Major clinical guidelines recommend definitive therapies, such as radical prostatectomy, for patients with a life expectancy of more than 10 years^6–8^. For reference, the average remaining life expectancy for a 75-year-old Japanese man is approximately 12 years, and for an 80-year-old man, it is approximately 9 years. These figures suggest that many elderly patients may still have sufficient longevity to benefit from curative treatment^9^. However, the decision-making process for older patients remains complex. Urologists must evaluate multiple factors, including life expectancy, comorbidities, baseline urinary function, and overall health status. Frailty has emerged as a critical concept for identifying “fit” versus “unfit” patients^10^. Several validated tools have been proposed for frailty assessment in geriatric oncology, including the Clinical Frailty Scale and the G8 screening tool. However, the tools for frailty assessment lack universal consensus, leaving much of the decision-making process to individual surgeons’ discretion.

The advent of robotic surgery, particularly robot-assisted radical prostatectomy (RARP), has expanded surgical indications for older patients due to its minimally invasive nature, reduced perioperative morbidity, enhanced precision, and favorable functional and oncological outcomes. Studies have demonstrated the feasibility of RARP in patients aged > 65 years, with acceptable oncologic and functional outcomes^11,12^. However, evidence regarding quality of life (QOL) outcomes after RARP in older adults, especially those aged ≥ 75 years, remains scarce^13–16^. Given the unique challenges faced by this population, including age-related physical and emotional vulnerabilities, assessing patient-reported outcomes (PROs) is crucial. Furthermore, perioperative complications in this age group require detailed evaluation to understand the balance between surgical risks and potential benefits. In addition, functional recovery, particularly urinary and sexual function, represents a major concern for elderly patients undergoing RARP.

This study aims to prospectively investigate QOL outcomes and evaluate perioperative complication rates in an age-stratified analysis of men undergoing RARP for PCa. By addressing these critical aspects, this research seeks to inform clinical decision-making and improve the management of PCa in the growing population of older adults.

## Results

### Study cohort and patient characteristics

Patients who consented to participate in the study were asked to complete a questionnaire, and a total of 619 individuals subsequently underwent RARP. Of these, 12 patients who failed to respond to at least two questionnaires were excluded from the analysis, along with three who withdrew their consent. Consequently, the final study cohort comprised 604 patients. The number of patients in the < 65, 65–74, and ≥ 75-year age groups was 143, 340, and 121, respectively. Patient characteristics are presented in Table 1. The number of comorbidities differed significantly among the three groups, with the ≥ 75-year group having the highest prevalence. Additionally, the rate of nerve-sparing procedures also showed a significant difference among the three groups, being highest in the < 65-year group. No other preoperative parameters exhibited statistically significant differences among the groups.

**Table 1:** Baseline patient characteristics.

Parameter	< 65 yr	65–74 yr	≥ 75	*p* value
	(*n* = 143)	(*n* = 340)	(*n* = 121)	
ASA-PS				0.117
PS1	33 (23.1)	67 (19.7)	24 (19.8)	
PS2	110 (76.9)	257 (75.6)	93 (76.9)	
PS3	0	16 (4.7)	4 (3.3)	
Number of comorbidities, median (range)	0 (0–2)	1 (0–3)	1 (0–3)	< 0.01
iPSA (ng/ml), median (range)	7.36 (1.47–70.00.47.00)	7.61 (2.94–78.97)	8.60 (2.73–88.3)	0.128
Clinical T-stage, n (%)				
T1	22 (15.4)	52 (15.3)	24 (19.8)	0.506
T2	105 (73.4)	243 (71.5)	76 (62.8)	
T3	16 (11.2)	44 (12.9)	20 (16.5)	
TX	0 (0.0)	1 (0.3)	1 (0.8)	
Biopsy Grade Group, n (%)				
1	22 (15.4)	62 (18.2)	18 (14.9)	0.361
2	45 (31.5)	119 (35.0)	32 (26.4)	
3	35 (24.5)	73 (21.5)	28 (23.1)	
4	28 (19.6)	59 (17.4)	24 (19.8)	
5	13 (9.1)	25 (7.4)	17 (14.0)	
NA	0 (0.0)	2 (0.6)	2 (1.7)	
Nerve-sparing surgery, n (%)	67 (46.9)	94 (27.6)	22 (18.2)	< 0.001

Data are presented as n (%) or median (range), unless otherwise indicated. Categorical variables were compared using the chi-square test, and continuous variables were compared using one-way analysis of variance (ANOVA) or the Kruskal–Wallis test, as appropriate. ASA-PS American Society of Anesthesiologists Physical Status classification system, PSA prostate-specific antigen. Grade Group was classified according to the ISUP grading system.

### Pathological oncologic features

Pathological oncologic features were comparable across the three age groups (Supplementary Table S1). There were no significant differences in pathological T stage, pathological N stage, pathological Grade Group, extraprostatic extension, positive surgical margin status, or seminal vesicle invasion.

### PROs

**Fig. 1:**
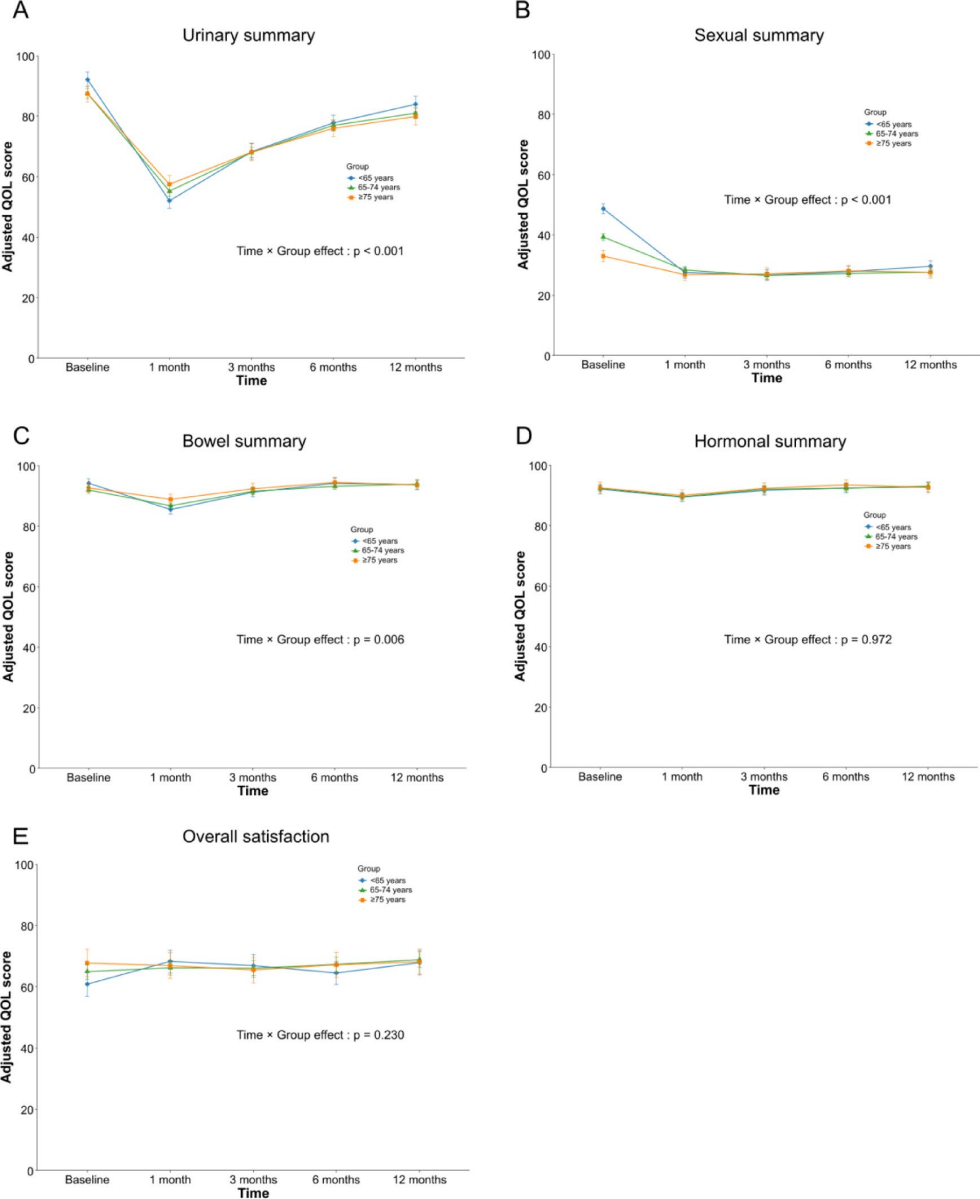
Longitudinal changes in EPIC domain scores after RARP by age group. Estimated marginal means of EPIC domain scores at baseline and at 1, 3, 6, and 12 months after RARP, stratified by age group (< 65, 65–74, ≥ 75 years). Panels show (**A**) urinary summary score, (**B**) sexual summary score, (**C**) bowel summary score, (**D**) hormonal summary score, and (**E**) overall satisfaction. Values are derived from linear mixed-effects models, with error bars indicating standard errors.

Longitudinal analyses of the Expanded Prostate Cancer Index Composite (EPIC) summary scores demonstrated domain-specific recovery patterns after RARP. For urinary, sexual, and bowel summary scores, a significant time-by-group interaction was observed, indicating that the trajectory of recovery varied by age. Younger patients (< 65 years) generally showed greater early postoperative decline, whereas older groups demonstrated more modest early changes (Fig. 1A,C). However, these differences diminished over time, and pairwise comparisons at 12 months showed no significant age-group differences in any of these domains (all adjusted p-values > 0.14). Hormonal summary scores and overall satisfaction showed no significant group effects or interactions, indicating comparable postoperative trajectories across age groups (Fig. 1D,E).

**Fig. 2:**
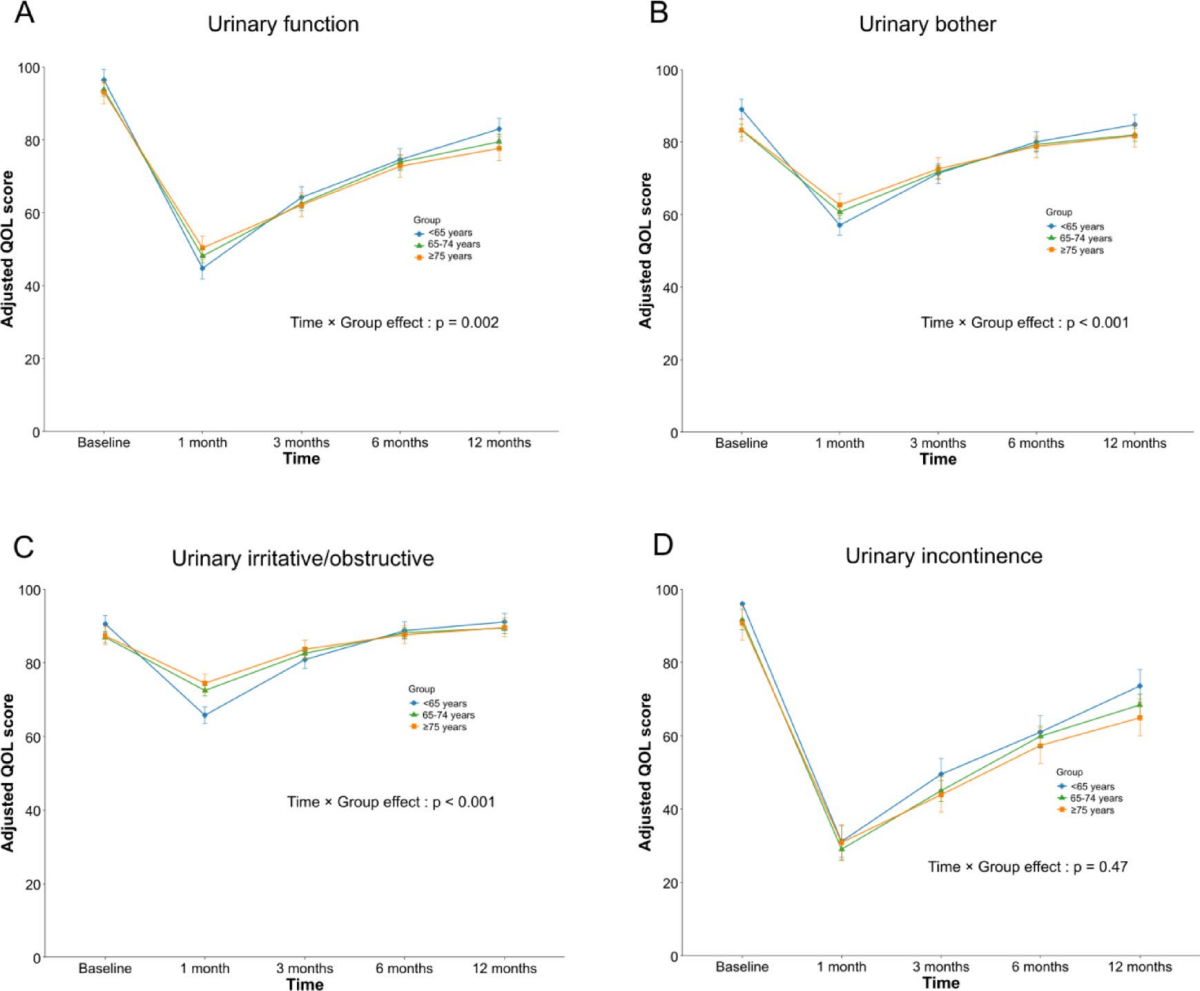
Longitudinal changes in urinary subdomain scores after RARP by age group. Estimated marginal means of urinary subdomain scores at baseline and at 1, 3, 6, and 12 months after RARP by age group (< 65, 65–74, ≥ 75 years). Panels show (**A**) urinary function, (**B**) urinary bother, (**C**) urinary irritative/obstructive, and (**D**) urinary incontinence scores. Values are derived from linear mixed-effects models; error bars indicate standard errors.

Urinary function declined markedly at 1 month in all age groups and gradually recovered thereafter. A significant time-by-group interaction was observed (*p* = 0.002), with slower recovery in men aged ≥ 75 years; however, no between-group differences were detected at baseline or 12 months, and only the < 65-year group showed lower scores than the ≥ 75-year group at 1 month (*p* = 0.031) (Fig. 2A). Urinary bother and irritative–obstructive scores also showed significant time-by-group interactions (both *p* < 0.001), but scores converged across age groups by 12 months (Fig. 2B,C). Urinary incontinence improved over time without a significant interaction (*p* = 0.47), although continence-related QOL at 12 months was slightly higher in men aged < 65 years than in those aged ≥ 75 years (adjusted *p* = 0.030) (Fig. 2D).

**Fig. 3:**
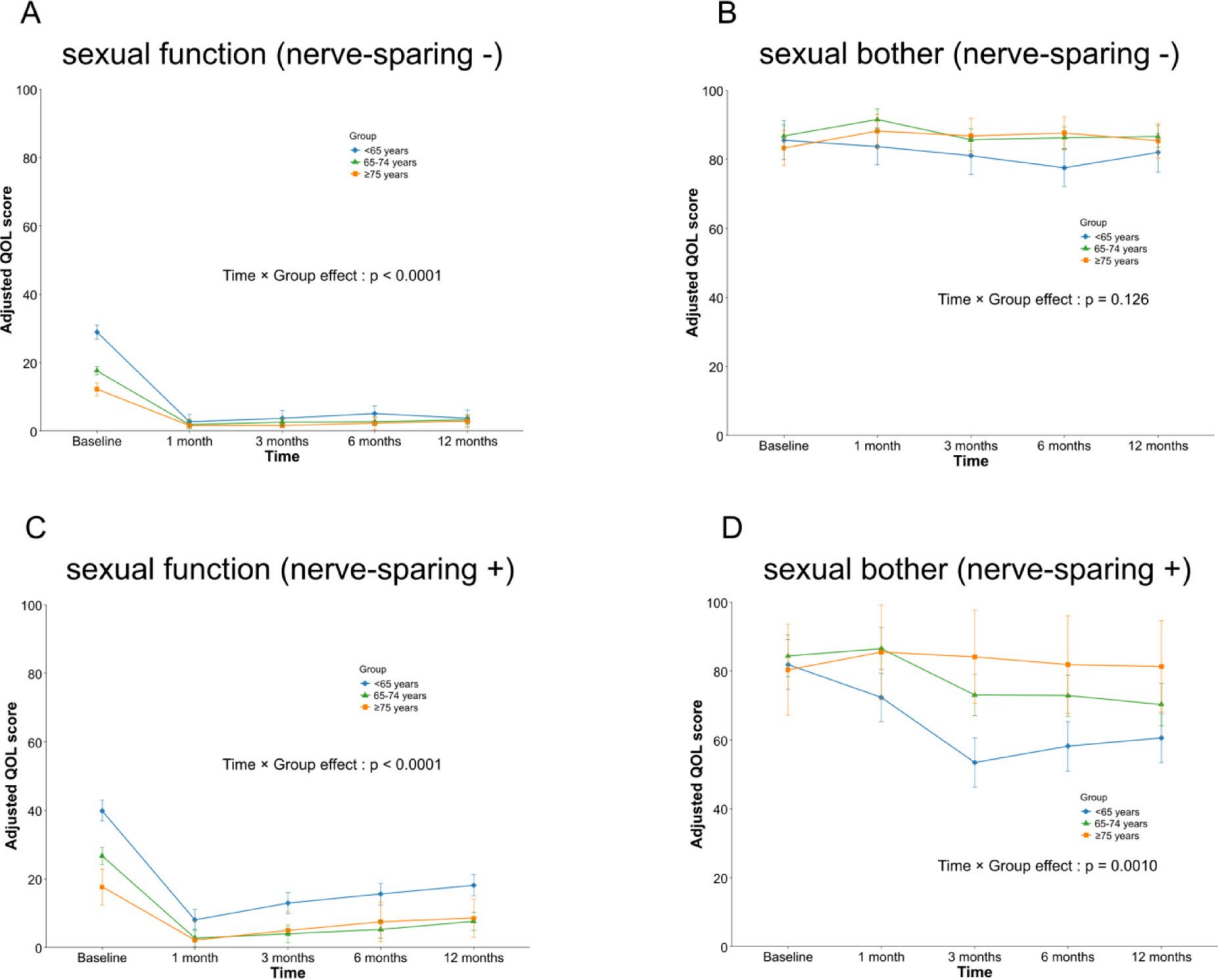
Longitudinal changes in sexual function and bother stratified by nerve-sparing status and age group. Estimated marginal means of sexual function and sexual bother scores at baseline and at 1, 3, 6, and 12 months after RARP, stratified by age group (< 65, 65–74, and ≥ 75 years) and nerve-sparing status. Panels show (**A**) sexual function without nerve-sparing, (**B**) sexual bother without nerve-sparing, (**C**) sexual function with nerve-sparing, and (**D**) sexual bother with nerve-sparing. Values are derived from linear mixed-effects models; error bars indicate standard errors.

Among patients without nerve-sparing surgery, sexual function decreased markedly at 1 month and remained uniformly low thereafter. Although younger men initially had higher baseline sexual function, postoperative scores did not differ across age groups (Fig. 3A). Sexual bother in this subgroup showed minimal temporal change and no significant age-group differences (Fig. 3B). In contrast, among patients who underwent nerve-sparing surgery, sexual function partially recovered over time and remained higher in younger men (< 65 years) (Fig. 3C), whereas sexual bother demonstrated a distinct age-dependent pattern, with minimal change in men aged ≥ 75 years and significantly higher bother-related QOL scores at 12 months compared with younger men (Fig. 3D). Sensitivity analyses treating age as a continuous variable using linear mixed-effects models demonstrated similar patterns for both urinary and sexual outcomes, supporting the robustness of the age-stratified analyses (Supplementary Table S2).

### Perioperative complications

Perioperative complications for each age group are listed in Table 2. There were no significant differences in the incidence of each complication among the three groups. Similarly, there were no significant differences among the three groups in the overall complication rates, either for any grade or for grade 3 or higher (*p* = 0.493 and *p* = 0.225, respectively).

**Table 2:** Perioperative complications within 30 days.

	Age <65 yr (n=143)			Age 65-74 yr (n=340)			Age ≥75 yr (n=121)			p value (any grade)	p value (grade≥3)
	Any grade	Grade <2	Grade≥3	Any grade	Grade <2	Grade≥3	Any grade	Grade <2	Grade≥3		
Bladder anastomotic leak	3	3	0	18	14	4	9	9	0	–	–
Hematuria	0	0	0	1	1	0	0	0	0	–	–
Urinary retention	1	1	0	1	1	0	0	0	0	–	–
Urinary tract infection	2	2	0	4	3	1	2	1	1	–	–
Lung infection	1	1	0	0	0	0	1	1	0	–	–
Pelvic abscess	0	0	0	2	0	2	0	0	0	–	–
Ileus	1	1	0	5	2	3	4	3	1	–	–
Gastrointestinal injury	0	0	0	1	0	1	0	0	0	–	–
Enterocolitis	0	0	0	2	1	1	0	0	0	–	–
Wound dehiscence	4	2	2	5	3	2	1	1	0	–	–
Wound infection	1	0	1	3	3	0	0	0	0	–	–
Thromboembolic event	0	0	0	1	0	1	0	0	0	–	–
Stroke	0	0	0	1	1	0	0	0	0	–	–
Arrhythmia	0	0	0	1	1	0	0	0	0	–	–
Lymph leakage	0	0	0	1	1	0	1	0	0	–	–
Elevated ALT	1	1	0	0	0	0	1	1	0	–	–
Postoperative hemorrhage	1	1	0	1	1	0	0	0	0	–	–
Total	15	12	3	47	32	15	19	16	2	0.439	0.62

Data are presented as n. Complications occurring within 30 days after surgery were identified from institutional clinical records and classified according to the Clavien–Dindo classification system. The diagnosis of complications was based on the judgment of each institution. p values were calculated using the chi-square test for comparisons among age groups.

## Discussion

This prospective multicenter study evaluated age-stratified functional outcomes and perioperative complications after RARP. Three principal findings emerged. First, overall urinary function recovery at 12 months was comparable across age groups, including men aged ≥ 75 years. Second, sexual function declined across all age groups irrespective of nerve-sparing status, yet sexual bother demonstrated a distinctly age-dependent pattern, with only minimal changes observed in men aged ≥ 75 years. Third, perioperative complication rates did not differ significantly among age groups, supporting the safety of RARP in appropriately selected elderly patients.

Urinary function showed substantial early postoperative decline followed by recovery, with no clinically significant age-group differences at 12 months. These findings are consistent with previous reports showing comparable urinary QOL recovery after RARP in older men^13^. Minor age-related differences in incontinence at 12 months—slightly favouring younger men—are consistent with earlier reports suggesting increased incontinence risk or slower recovery in older adults^11,14,17,18^. Baseline urinary function has also been associated with postoperative continence outcomes, suggesting that patient selection based on baseline urinary function may be important in elderly patients^19^. Notably, younger patients (< 65 years) exhibited a more pronounced decline at 1 month in urinary irritative/obstructive and urinary bother scores compared with older age groups, although these differences diminished over time and scores converged by 12 months. This finding suggests that younger men may experience greater subjective burden from early postoperative lower urinary tract symptoms, even when long-term recovery is comparable across age groups. From a clinical perspective, incorporating this age-specific early trajectory into preoperative counseling may help align expectations and improve early postoperative satisfaction, particularly among younger patients who may be more sensitive to temporary symptom worsening. Our results highlight that age-related differences after RARP are not limited to continence recovery alone but may also involve early postoperative irritative/obstructive symptoms and associated bother, which have been less emphasized in prior age-stratified PRO analyses.

Sexual function decreased universally after RARP, reflecting the effects of neurovascular bundle resection in non–nerve-sparing procedures and inadvertent partial nerve injury during nerve-sparing surgery. Among men undergoing nerve-sparing procedures, younger patients maintained higher sexual function scores, likely reflecting better baseline function. In contrast, sexual bother showed an age-related pattern, with greater postoperative changes in younger men but minimal change in men aged ≥ 75 years. This dissociation between functional decline and subjective bother suggests that postoperative adaptation may differ by age, potentially influenced by preoperative expectations^20^. Similar findings of limited sexual function recovery in elderly patients have been reported^15^. Compared with previous studies, our study provides additional insights by evaluating longitudinal trajectories of PROs in a prospective multicenter cohort, with specific focus on patients aged ≥ 75 years, a population that has been underrepresented in prior reports. Importantly, we also evaluated PRO trajectories according to nerve-sparing status, allowing age- and nerve-sparing–stratified analyses of postoperative sexual function and sexual bother. Notably, although sexual function declined across all age groups, sexual bother showed minimal change in men aged ≥ 75 years, suggesting age-related differences in psychological adaptation after RARP.

Perioperative complication rates did not differ significantly among age groups. This finding aligns with prior studies demonstrating the feasibility and safety of RARP in elderly men, including those aged ≥ 75 years^13,21–23^. Although some reports have suggested a modest increase in complications with age, the clinical impact appears limited in well-selected patients^24^. The inclusion of a large cohort of older men strengthens the evidence supporting RARP as a safe surgical option in appropriately selected elderly patients.

These findings have practical implications for counseling and shared decision-making. Age alone should not preclude consideration of RARP in men aged ≥ 75 years, as urinary recovery and perioperative safety were comparable to younger patients. A subset of patients may have elected to undergo RARP despite having low-risk disease because of pre-existing urinary difficulties or concerns about biopsy undergrading and disease progression during active surveillance. Recent evidence has demonstrated the feasibility and safety of RARP even in octogenarian men. Shahait et al. reported favorable perioperative and functional outcomes in patients aged 80 years and older, with acceptable continence recovery and complication rates in a multi-institutional setting^16^. In addition, the age-dependent pattern in sexual bother highlights the need for individualized counseling, particularly for younger men who may experience greater distress related to postoperative sexual changes.

Although the prospective multicenter design is a strength, several limitations should be acknowledged. First, the relatively short follow-up of one year limits the evaluation of long-term functional recovery. Second, because elderly patients undergoing surgery are typically carefully selected and relatively fit, and because the study population consisted exclusively of Japanese patients, selection bias may have been present, limiting the generalizability of the findings to the broader elderly population. Third, long-term oncological outcomes such as PSA persistence and biochemical recurrence were not collected in the original study protocol, as the study primarily focused on perioperative safety and patient-reported functional outcomes. Therefore, further studies with longer follow-up are needed to clarify oncological outcomes in elderly patients undergoing RARP. Fourth, comorbidity burden was assessed using the number of comorbidities rather than a validated index such as the Charlson Comorbidity Index (CCI), as the data required to calculate the CCI were not available. In addition, nerve-sparing status was classified dichotomously without detailed grading of nerve preservation, which may have influenced functional recovery outcomes.

In conclusion, RARP can be safely performed in well-selected patients aged ≥ 75 years, with postoperative urinary and overall QOL recovery comparable to younger men.

## Methods

### Study design and participants

This investigation was structured as a multi-institutional, prospective observational study. The cohort included men diagnosed with prostate cancer who received primary treatment via RARP between January 2020 and December 2022. Written informed consent was obtained from all participants prior to their inclusion. Patients who did not complete more than half of the postoperative questionnaires were excluded from the study. Additionally, those who expressed a desire to withdraw from the study during the research period were also excluded. A flow diagram of patient inclusion is provided in Supplementary Figure S1. Patients aged 44–84 years were enrolled and stratified into three age groups: <65 years, 65–74 years, and ≥ 75 years. Nerve-sparing procedures were categorized as present or absent based on the surgical records. Perioperative management was performed according to a protocol of each institution. Physical prophylaxis was used to prevent deep vein thrombosis, while pharmacologic prophylaxis was not routinely administered.

### Outcome measures

Patient-reported outcomes were assessed using the Expanded Prostate Cancer Index Composite (EPIC-50) at baseline and at 1, 3, 6, and 12 months after RARP. The EPIC questionnaire evaluates five key health-related quality-of-life domains: urinary summary score, bowel summary score, sexual summary score, hormonal summary score, and satisfaction, each scored on a scale from 0 to 100. Each domain was evaluated comprehensively because it is a standard component. Urinary incontinence was evaluated using the urinary incontinence subdomain of the EPIC questionnaire. The hormonal domain was included as it is a standard component of the EPIC questionnaire used to comprehensively evaluate patient-reported outcomes after prostate cancer treatment. Perioperative complications occurring within the first month following surgery were identified from institutional clinical records at each participating center and classified according to the Clavien-Dindo classification system. The diagnosis of complications was based on the judgment of each institution. Long-term oncological outcomes, such as PSA persistence and biochemical recurrence, were not included in the original study protocol, as the primary objective of this study was to evaluate perioperative safety and longitudinal patient-reported functional outcomes after RARP.

### Statistical analysis

Baseline patient characteristics were compared among age groups using the chi-square test for categorical variables and analysis of variance (ANOVA) or the Kruskal–Wallis test for continuous variables, as appropriate. Longitudinal changes in EPIC scores were analyzed using linear mixed-effects models including time, age group, and their interaction as fixed effects, with patient ID as a random intercept. When a significant interaction between time and age group was detected, post-hoc pairwise comparisons were performed using estimated marginal means (EMMs) with Tukey adjustment. Perioperative complications occurring within 30 days after surgery were compared among age groups using the chi-square test. All tests were two-sided, and p-values < 0.05 were considered statistically significant. Analyses were conducted using R (version 4.0.3). EMMs and their standard errors (SE) for each age group at each postoperative time point were derived from the mixed-effects models and are presented in Supplementary Tables S3–S5.

## Supplementary Information

Below is the link to the electronic supplementary material.

Supplementary Material 1

Supplementary Material 2

## Data Availability

The datasets generated and/or analyzed during the current study are available from the corresponding author on reasonable request.

## References

[1] Bray, F. et al. Global cancer statistics 2022: GLOBOCAN estimates of incidence and mortality worldwide for 36 cancers in 185 countries. *CA Cancer J. Clin.* **74**, 229–263 (2024). 38572751 10.3322/caac.21834

[2] Kontis, V. et al. Future life expectancy in 35 industrialised countries: projections with a Bayesian model ensemble. *Lancet*. **389**, 1323–1335 (2017). 28236464 10.1016/S0140-6736(16)32381-9 PMC5387671

[3] Owens, L. et al. Trends in age and prostate-specific antigen at prostate cancer diagnosis between 2010 and 2019. *JNCI Cancer Spectr.* **8**, pkae106 (2024). 39441819 10.1093/jncics/pkae106 PMC11578289

[4] Okuyama, A. & Higashi, T. Patterns of cancer treatment in different age groups in Japan: An analysis of hospital-based cancer registry data, 2012–2015. *Jpn. J. Clin. Oncol.* **48**, 417–425 (2018). 29590399 10.1093/jjco/hyy032

[5] World Health Organization Life expectancy at birth.https://www.who.int/data/gho/data/indicators/indicator-details/GHO/life-expectancy-at-birth-(years).

[6] National Comprehensive Cancer Network. *NCCN Clinical Practice Guidelines in Oncology: Prostate Cancer* Version 5. https://www.nccn.org/professionals/physician_gls/pdf/prostate.pdf (2026). 10.6004/jnccn.2026.0001 41671464

[7] Cornford, P. et al. Guidelines on prostate cancer—2024 update. Part I: screening, diagnosis, and local treatment with curative intent. *Eur. Urol.* **86**, 148–163 (2024). 38614820 10.1016/j.eururo.2024.03.027

[8] Eastham, J. A. et al. Clinically localized prostate cancer: AUA/ASTRO guideline, Part I. *J. Urol.* **208**, 10–18 (2022). 35830561 10.1097/JU.0000000000002854

[9] Ministry of Health. Labour and Welfare, Japan. *Abridged Life Tables for Japan* https://www.mhlw.go.jp/toukei/saikin/hw/life/life24/index.html (2024).

[10] Shahait, M. et al. A 5-Item frailty index for predicting morbidity and mortality after radical prostatectomy: an analysis of the american college of surgeons national surgical quality improvement program database. *J. Endourol*. **35**, 483–489 (2021). 32935596 10.1089/end.2020.0597

[11] Holze, S. et al. Age-stratified outcomes after radical prostatectomy in a randomized setting (LAP-01). *World J. Urol.* **40**, 1151–1158 (2022). 35124734 10.1007/s00345-022-03945-0 PMC9085667

[12] Traboulsi, S. L. et al. Functional and perioperative outcomes in elderly men after robotic-assisted radical prostatectomy for prostate cancer. *World J. Urol.* **38**, 2791–2798 (2020). 32034499 10.1007/s00345-020-03096-0

[13] Togashi, K. et al. Oncologic and patient-reported outcomes after robot-assisted radical prostatectomy in men aged ≥ 75 years. *Urol. Oncol.* **39**, 729e17–729e25 (2021). 10.1016/j.urolonc.2020.12.001 33353866

[14] Gondoputro, W. et al. How does age affect urinary continence following robot-assisted radical prostatectomy? *J. Urol.* **207**, 1048–1056 (2022). 34978202 10.1097/JU.0000000000002391

[15] Leyh-Bannurah, S. R. et al. Feasibility of robot-assisted radical prostatectomy in men aged ≥ 75 years. *Aging Male*. **25**, 8–16 (2022). 34957914 10.1080/13685538.2021.2018417

[16] Shahait, M. et al. Perioperative and functional outcomes of robot-assisted radical prostatectomy in octogenarian men. *J. Endourol*. **35**, 1025–1029 (2021). 33267679 10.1089/end.2020.0859

[17] Yamada, Y. et al. Comparison of perioperative outcomes in elderly (age ≥ 75 years) vs. younger men undergoing robot-assisted radical prostatectomy. *PLoS ONE*. **15**, e0234113 (2020). 32497131 10.1371/journal.pone.0234113 PMC7272059

[18] Koss Modig, K. et al. Patient- and procedure-specific risk factors for urinary incontinence after robot-assisted radical prostatectomy: a nationwide, population-based study. *Eur Urol Oncol* **8**, 932–940 (2025). 40307091 10.1016/j.euo.2025.03.015

[19] Majima, T. Urodynamic evaluation before and after RARP to identify pre- and intraoperative factors affecting postoperative continence. *Neurourol. Urodyn.* **40**, 1147–1153 (2021). 33846995 10.1002/nau.24650

[20] Hampson, L. A. et al. Impact of age on quality-of-life outcomes after treatment for localized prostate cancer. *Eur. Urol.* **68**, 480–486 (2015). 25656807 10.1016/j.eururo.2015.01.008

[21] Koterazawa, S. et al. Effects of aging on complications following robot-assisted radical prostatectomy. *Int. J. Clin. Oncol.* **30**, 340–350 (2025). 39621176 10.1007/s10147-024-02660-7

[22] Ubrig, B., Boy, A., Heiland, M. & Roosen, A. Outcome of robotic radical prostatectomy in men over 74. *J. Endourol*. **32**, 106–110 (2018). 29232985 10.1089/end.2017.0512 PMC5813731

[23] Carbin, D. D. et al. Robot-assisted radical prostatectomy in Indian men aged ≥ 75 years: a propensity score-matched analysis. *J. Robot Surg.* **16**, 799–806 (2022). 34455530 10.1007/s11701-021-01301-9

[24] Zhu, A. et al. Effect of age on robotic-assisted radical prostatectomy outcomes: a multicenter analysis. *Urology* **202**, 78–84 (2025). 40349786 10.1016/j.urology.2025.05.007 PMC13051560

