# Supplementary figures and images for: Age-stratified trajectories of patient-reported outcomes and perioperative safety after robot-assisted radical prostatectomy: a prospective multicenter cohort study
Source: Sci Rep. 2026 Mar 31;16:15241. doi: 10.1038/s41598-026-46171-z (PMC13181070; doi:10.1038/s41598-026-46171-z)

Supplementary Figure S1. Flow diagram of patient selection and inclusion in the study cohort.

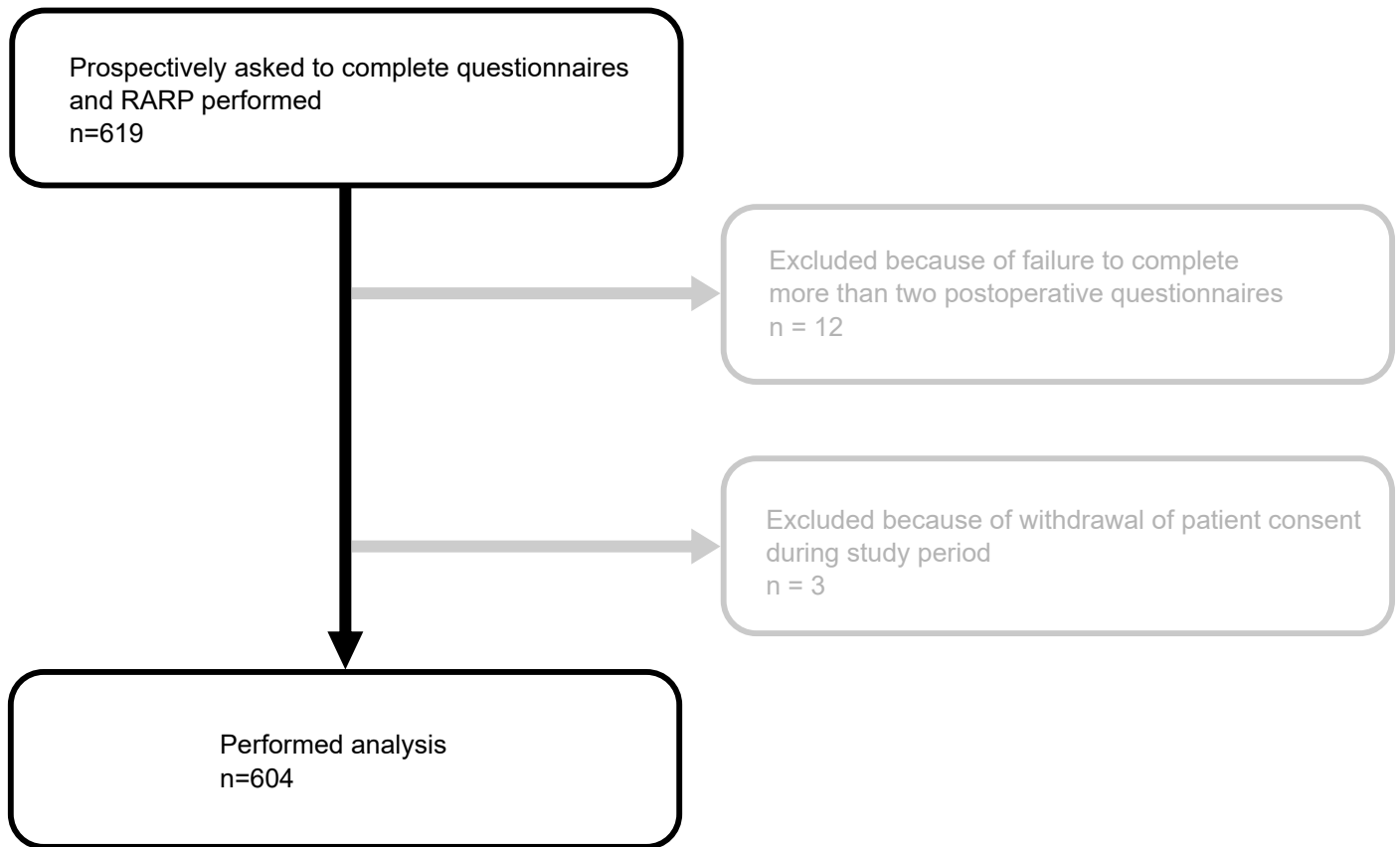

Supplement: Supplementary file 2 — Supplementary Material 2 [file 41598_2026_46171_MOESM2_ESM.pdf]
